# Insights into Gut Dysbiosis: Inflammatory Diseases, Obesity, and Restoration Approaches

**DOI:** 10.3390/ijms25179715

**Published:** 2024-09-08

**Authors:** Andy Acevedo-Román, Natalia Pagán-Zayas, Liz I. Velázquez-Rivera, Aryanne C. Torres-Ventura, Filipa Godoy-Vitorino

**Affiliations:** Microbiology Department, University of Puerto Rico Medical Sciences Campus, San Juan 00936, Puerto Rico

**Keywords:** gut microbiota, obesity, ulcerative colitis, Crohn’s disease, *Clostridioides difficile*

## Abstract

The gut microbiota is one of the most critical factors in human health. It involves numerous physiological processes impacting host health, mainly via immune system modulation. A balanced microbiome contributes to the gut’s barrier function, preventing the invasion of pathogens and maintaining the integrity of the gut lining. Dysbiosis, or an imbalance in the gut microbiome’s composition and function, disrupts essential processes and contributes to various diseases. This narrative review summarizes key findings related to the gut microbiota in modern multifactorial inflammatory conditions such as ulcerative colitis or Crohn’s disease. It addresses the challenges posed by antibiotic-driven dysbiosis, particularly in the context of *C. difficile* infections, and the development of novel therapies like fecal microbiota transplantation and biotherapeutic drugs to combat these infections. An emphasis is given to restoration of the healthy gut microbiome through dietary interventions, probiotics, prebiotics, and novel approaches for managing gut-related diseases.

## 1. The Importance of the Gut Microbiota for Human Health

The human gut microbiota is a complex community of microorganisms that co-evolved with us and reside in the gastrointestinal tract. This co-evolution with human cells was done in the context of many changes, including lifestyle, such as medications, urbanization, and dietary habits. The gut microbiome includes bacteria, archaea, relatively less-known fungi, and viruses. Bacteria are well surveyed and are the focus of our review. The development of high-throughput DNA and RNA sequencing technologies, along with advanced computational methodologies, has allowed scientists to catalog microorganisms comprehensively in an unprecedented manner. Various body habitats host distinct microbial communities and microbiomes that vary in microbial composition and function, including metabolic modules and pathways [1]. We now know that each body site has its own distinct composition of microbial communities depending on the physiological characteristics of the body niche [2]. Poor diet, antibiotic use, infections, and stress can lead to dysbiosis, characterized by a loss of beneficial microbes, microbial diversity, and the appearance of harmful bacteria.

The biogeography of the gut compartments (small and large intestines) affects the composition of the microbiota. Following the upper digestive tract, like the oral cavity and esophagus, the duodenum and jejunum have lower diversity than the ileum or the proximal colon [3]. The small intestine is predominantly populated by Gram-positive Firmicutes, including genera such as *Streptococcus*, *Veillonella*, and *Clostridium* [3]. In contrast, the colon hosts different dominant bacterial groups, especially strict anaerobes. The distal small intestine has higher abundances of bacterial phyla such as Bacteroidetes and Proteobacteria. The large intestine, composed of the cecum, ascending, transverse, descending, and sigmoid colon, rectum, and anus, harbors rich and highly diverse microbial communities, primarily consisting of two bacterial phyla in healthy individuals: Bacteroidetes and Firmicutes. At the genus level, *Prevotella*, *Clostridium*, *Lactobacillus*, *Ruminococcus*, or *Odoribacter* [4], as well as *Roseburia*, *Faecalibacterium prausnitzii*, *Bacteroides vulgatus*, or *Fusicatenibacter saccharivorans*, are reduced in colon cancer and important anti-inflammatory taxa of the distal gut [5]. Various intestinal bacteria transform simple sugars into organic acids like lactate, acetate, propionate, and butyrate (short-chain fatty acids (SCFAs), which impact the proliferation and virulence of pathogens. For instance, acetate produced by *Bifidobacterium* spp. can suppress the virulence of Shiga-like toxins from verotoxigenic *E. coli* [6,7]. Dysbiosis has been implicated in several gastrointestinal disorders, including inflammatory bowel disease (IBD) [8], irritable bowel syndrome (IBS) [9], and other problems including *Clostridioides difficile* infections. For instance, patients with IBD often show reduced levels of beneficial bacteria like *Faecalibacterium prausnitzii* and increased levels of pro-inflammatory microbes. Beyond the gut, dysbiosis is linked to systemic conditions such as obesity [10] and even neurological disorders like depression and anxiety [11], and often, dietary changes and probiotic consumption help improve mood and overall health.

Restoring a healthy gut microbiome to counteract dysbiosis involves dietary interventions, probiotics, prebiotics, and fecal microbiota transplantation (FMT). Dietary changes, such as increased fiber intake, can promote the growth of beneficial bacteria. Below are some specific cases where microbiota modulation has been proven to improve phenotypic and symptomatic outcomes within gut-microbiome-related problems.

## 2. Inflammatory Bowel Diseases: Ulcerative Colitis and Crohn’s Disease

### 2.1. Crohn’s Disease Etiology and Pathogenesis

Crohn’s disease is a chronic gastrointestinal inflammatory condition that can affect a patient’s digestive system, anywhere from the mouth to the anus. By generating an exaggerated immune response throughout the digestive tract in inconsistent patches of tissue inflammation, it demonstrates symptoms such as abdominal pain, diarrhea, bleeding, nausea and vomiting, loss of appetite, weight loss, and fatigue [12]. These symptoms all depend on the location of the disease and the host’s respective immune response, meaning that the condition can vary greatly between patients, and having an equal chance of affecting males and females aged between 15 and 35. Prolonged inflammatory activity along the digestive tract can lead to disease aggravation, further resulting in strictures (narrowing of the gut wall due to scar tissue), abscesses, and fistulas [13]. In 2019, both inflammatory bowel diseases (IBDs), Crohn’s disease and ulcerative colitis, were reported to have a prevalence of approximately 24.51, 20.03, and 16.94 out of 100,000 people in North America, Australasia, and Western Europe, respectively [14]. The mechanisms by which this disease originates in a person are still unknown. Still, research indicates genetic, immunological, and environmental factors contribute to the onset of these intestinal perturbations [15].

**Figure 1:**
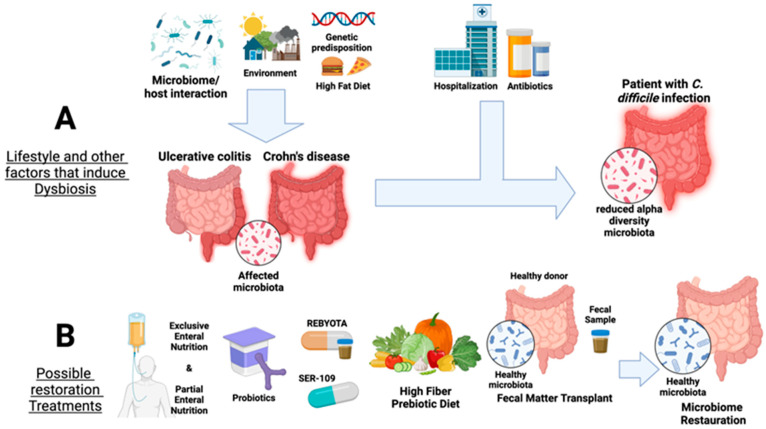
Overview of factors that affect the gut microbiota and current available therapies for IBD, namely ulcerative colitis and Crohn’s disease, as well as in *Clostridioides difficile* infection. Panel (**A**) summarizes the factors that induce dysbiosis in the microbial gut communities, such as microbiome/host interaction, environmental factors, high-fat diet, and genetic predisposition leading to a reduction in gut alpha diversity. Panel (**B**) shows available microbiota restoration therapies for UC and CD and treatments against *C. difficile* colonization (probiotic supplementation, Rebyota/SER-109 Partial Enteral Nutrition, high-fiber prebiotic diet, and fecal microbiota transplants). Created with Biorender.

Modern lifestyles have been well linked with the development of inflammatory diseases (Figure 1A). The Western lifestyle brings about practices such as cigarette smoking, antibiotic usage, and reduced exposure to microbes throughout childhood by urbanization and hygiene practices, which are known to increase the risks of developing Crohn’s disease [16]. Recently an important study has shown how rural people in China and Israel shift their microbiota and metabolic profiles towards a Crohn’s disease profile in urban settings [17]. The study compared the microbiome and metabolome of healthy and CD patients in developing rural and modern urban Chinese communities with a Westernized Israeli population. In rural settings, higher exposure to bacteria is shown with the presence of cattle and farms, extended family cohabitation, less hygiene, and a less processed diet that incorporates more vegetables and fruits. Meanwhile, the opposite is observed in urban settings, showing that the reduction in microbial diversity correlates with increased disease incidence. It demonstrated the significant impact of a Western diet involving high fats and processed foods on the gut microbial taxa, showing an increase in communities such as Fusobacteriaceae-like taxa linked with the Crohn’s disease profile [17].

### 2.2. Clinical Significance of Crohn’s Disease and Diagnostic Procedures

There is no straightforward screening method for Crohn’s disease. The current analysis detects hematological biomarkers, such as C-reactive protein (CRP) and clinical history. CRP is produced by the liver and used as a marker of inflammation. Testing for CRP is used to try to distinguish between types of IBD conditions. Another way to survey disease progression is by studying microbiota metabolites, which are molecule end-products of metabolic processes, such as peptides and lipids [18]. Among some of the important metabolic markers are loss of SCFAs, and an increase in bile acid, fecal amino acid, and lipopolysaccharide (LPS) levels in blood, the functions of which can regulate microbial communities and maintain homeostasis of gut bacteria. These metabolites are actively present in healthy intestinal flora compared to the dysregulated quantities found in Crohn’s patients [19]. Metabolites, such as bile acids, can be used to understand the presence of commensal gut bacteria and how disorders in these types of intestinal acids can negatively impact the composition of microbes present in this system. Lipopolysaccharides in high quantities are known to induce chronic inflammation. With damage to gut tissues and permeability, these molecules then enter the bloodstream, cause systemic inflammation, and can further progress IBD [20]. LPS triggers key immune system components, such as TLR4, which stimulates the NFkB pathway known for inducing inflammatory responses and activates other molecules like IFN-γ and TNF-α, which further enhance inflammation pathways. SCFAs, including acetate, propionate, and butyrate, are produced by gut bacteria, like Bacteroidetes, that select for and ferment dietary fiber and prebiotics as a source of energy for the host [21]. These SCFAs reinforce intestinal barrier functions, maintain proper immune responses with regulatory T cells, and promote gut microbial homeostasis and mucosal tissue regeneration [22]. These hematological and metabolic molecules that modulate diverse immune responses and gut functions are key biomarkers in CD diagnostics.

Dysbiosis in the microbiota can lead to many negative changes, as the gut plays a role in the developing immune system. In addition, the usage of antibiotics has been repeatedly shown to have a detrimental impact on the intestinal microbiota by allowing the displacement of a healthy flora (Table 1). An example of these circumstances can be seen in escalating health conditions such as cancer, rheumatoid arthritis, cardiovascular diseases, and obesity. This is a result of gut microbiota disturbances that can diminish the colonization resistance of beneficial commensal bacteria and provide an opportunity to pathogenic microbes residing in the intestines to harm the digestive system [23]. With the rising prevalence of disease, disrupting symptoms of health, and complicated origin pathways, it is highly important to provide viable treatments for these patients. A cure for Crohn’s disease is currently unknown. Meanwhile, present treatments offer ways to handle and cope with the symptoms. Anti-inflammatory medication such as corticosteroids can mainly reduce swelling and inflammation in the digestive tract. Recently, biologic agents have been more readily implemented as lower-risk medication. These medication types only suppress certain gut immune system components, such as interleukins, integrins, and TNF, avoiding inhibiting other useful immune pathways [12]. However, even with a concurrent and stable treatment, surgery procedures may be required in more advanced cases due to the formation of bowel obstructions or strictures [12].

### 2.3. Treatment Options for Crohn’s Disease

One recommended method for treating these conditions is the use of probiotics, as they have been shown to promote a healthier intestinal flora, inducing remission, and maintaining the remission period in Crohn’s disease patients. The predominant presence of bacteria such as *Bacteroides*, *Faecalibacterium*, *Bifidobacterium*, and *Lactobacillus* species have been proposed as beneficial in maintaining a colonized gut, augmenting gut microbial diversity in a reduced environment, and improving other organ functions affected by dysbiosis [24,25]. Studies have found that implementing the use of probiotics decreases inflammation markers throughout the usage period, presents less-recurrent flare-ups, and maintains more extended remission periods, keeping symptoms at bay [24]. However, further studies need to be conducted to better identify a proper probiotic and prebiotic treatment plan, since not all studies present reproducible and concurrent data. Below, we summarize several published approaches shown to improve gut symptoms. To reinforce the usage of probiotics, supplementing with a proper precise diet can help combine individual benefits. Exclusive enteral nutrition (EEN) is a fully liquid-based diet, mainly aimed at children, to bypass any issues with nutrient absorption and benefit weight gain, facilitating gut mucosal healing [26]. Many reports in the last 30 years indicate high remission rates above 70% in various studies implementing EEN in children, concordant with clinical medication effects, demonstrating significant similarity in anti-inflammatory treatment while being less impactful on the body [26]. Solid foods are slowly incorporated at the end of the treatment period to avoid the return of symptoms, although this is not always successful. Meanwhile, a partial enteral nutrition (PEN) method has recently been used to incorporate a half-liquid/half-solid diet with higher tolerance rates than a liquid-only diet. A final significant therapy under active clinical studies and pending FDA approval in Crohn’s disease patients is the fecal microbiota transplant (FMT); this is mainly seen in a pediatric setting to treat mild to moderate disease early on [27,28]. Details of this procedure and other prebiotic and probiotic restoration methods are provided in Section 5.

### 2.4. An Overview of Ulcerative Colitis and the Microbiota

Ulcerative colitis (UC) is an autoimmune inflammatory disease that targets the large intestine and progresses gradually. It can be diagnosed at any age and increases the risk of developing colon cancer by 4.5% after 20 years of diagnosis [29]. Still, it is more common in people aged 15 to 30 years [30]. Factors including microbiome/host interactions, the environment, and genetic predisposition can affect the pathogenesis of this disease [31]. The clinical manifestations include weight loss, bloody stool, diarrhea, shortened colon, and inflammatory infiltration [32]. The World Health Organization has cataloged UC as one of the most complex diseases to treat nowadays [32].

Previous research has demonstrated a decrease in microbial diversity and an alteration of homeostasis associated with UC [33,34]. Studies found a significant reduction in the Phylum Firmicutes, and an increase mostly in Proteobacteria, but also in some members of Bacteroidetes [35,36]. Within Firmicutes, an important loss of *Roseburia*, *Faecalibacterium* [33,37,38], and *Lactobacillus* [39,40] is notorious, as these protective taxa are key for maintaining a healthy gut (Table 1). Studies related to bacterial and fungi interactions could uncover more details about the role of the microbiome in a UC profile. A study conducted by Sovran et al. in 2018 investigated the relationship between fungi and certain intestinal bacteria that play a role in the development of UC. *S. boulardii* and *C. albicans*, which can have either beneficial or detrimental effects, respectively, depending on the bacterial diversity within the gut microbiome [41].

Different species of *Lactobacillus* are associated with a positive reduction in the severe symptoms provoked by this syndrome, like colon shortening and rectal bleeding [39]. Species like *Lactobacillus johnsonii* can produce anti-inflammatory metabolites resulting in the induction of macrophage M2-mediated responses. This leads to an enhancement of the production of IL-10, an anti-inflammatory cytokine [40]. *Lactobacillus acidophilus* helps support the intestinal barrier, reduces the magnitude of proinflammatory cytokines, and enhances the production of acetate [42]. Likewise, *Lactobacillus casei* triggers an anti-inflammatory reaction by suppressing the pro-inflammatory response and helping restore the intestinal barrier [32]. Additionally, *Bifidobacterium*, a genus from the phylum Actinobacteria, is downregulated. *Bifidobacterium lactis* (BLa80) helps to reduce excessive weight loss, colon shortening, and inflammation [38,43]. Research has demonstrated that *B. Lactis* species can restore homeostasis [44]. *Faecalibacterium prausnitzii* can similarly help reduce symptoms produced by UC, as it increases the length of the colon and reduces inflammatory cytokines in a pre-clinical model [45]. Additionally, the *Faecalibacterium* genus produces large amounts of butyrate, the primary energy source for colonocytes—the epithelial cells on the surface of the colon—aiding in the restoration of these cells [46].

Regarding phyla that are upregulated, it is crucial to highlight the increase in the Bacteroidetes because approximately 50% of the proteins secreted by microorganisms related to UC are secreted or derived from the genus of *Bacteroides*. *Bacteroides* can generate proteases which are correlated with proteolysis, aggravating the severity of ulcerative colitis. Remarkably, *Bacteroides vulgatus* and *Bacteroides dorei* produce proteases that break down serine and/or cysteine [47], and these enzymes are correlated with the initiation of this illness. A study has proposed the inhibition of the indicated serine proteases produced by *Bacteroides vulgarus* as a treatment for UC because approximately 40% of the patients with ulcerative colitis are prone to have these bacteria expressing serine proteases in large amounts, and this can be one of the reasons for the disruption of the colon [47]. Moreover, *Enterobacteriaceae* within phylum Proteobacteria have been observed in more significant amounts during inflammatory bowel diseases, especially the genera *Escherichia*, *Klebsiella*, and *Citrobacter* (Table 1) [43,48]*. Escherichia coli* is a commensal microorganism that, depending on the conditions and immune system of the host, can switch to being a harmful pathogen capable of triggering inflammation in the host [48]. It can also produce serine proteases enzymes that have been previously described as harmful in the case of *Bacteroides* [49]. In addition, studies in mice have shown that *Citrobacter rodentium* decreases P-glycoprotein transporters in the colon; these are efflux transporters that serve to export out of the cell toxic compound. A decrease in this molecule can induce an accumulation of toxins in the intestinal epithelial cells that can later be a factor inducing the alteration of the epithelial integrity and the gut microbiota [50]. Furthermore, other factors like inflammatory cytokines negatively affect the expression of P-glycoprotein transporters [50]. Indeed, it is important to mention that in both mice and humans, gut dysbiosis is characterized by an imbalance in the microbiota (share in ~90% in both groups), and changes in flora are closely associated with the development and progression of inflammatory diseases. 

Depending on the species and conditions, some metabolites are secreted or generated by microorganisms. Amino acid metabolites like Glutamine and Arginine have been demonstrated to be at higher levels in inflammatory bowel diseases [38]. A study conducted in pre-clinical models (rats) has revealed that the administration of Evodiamine—a quinolone alkaloid extracted from a Chinese tree—can help to reduce the levels of Glutamine in the serum and enhance the beneficial microbiota of the gut that are downregulated in UC [42]. The precise mechanism has not been elucidated, but the article indicated that a treatment using this quinolone can increase levels of *Lactobacillus*, specifically *Lactobacillus acidophilus*, which previously has been described as probiotic gut bacteria [42]. Furthermore, Evodiamine can downregulate IL-1β and IL-6 cytokines (pro-inflammatory), and upregulate IL-10 (anti-inflammatory), in experimental models with induced UC [42,51]. Lastly, metabolic products like pyruvic acid, fumaric acid, malonic acid, and oxoglutaric acid have been detected at higher levels in models with UC [38]. It is suggested that the increased energy production can cause the increase in pyruvic acid via glycolysis in hypoxic conditions induced by these diseases. This, in turn, leads to the accumulation of pyruvates that cannot be used in the tricarboxylic acid cycle [52], and in hypoxic conditions, an excess of pyruvate can induce an overflow of lactate, which causes an acidic environment and dysbiosis [53]. Understanding these metabolic changes can be crucial in determining potential targets for therapeutic intervention to treat UC.

Due to the large number of individuals affected by UC nowadays, there is a constant search for an effective treatment that enables these individuals to lead normal lives. An approved treatment is Vedolizumab, a monoclonal antibody that targets α4β7 integrin expressed on the gut lymphocyte surface [54]. This action inhibits the migration of these cells to the gut, thereby reducing inflammation [54]. Scientists are searching for a treatment beyond that of modulating the host’s immune response but instead focusing on the microbiome, and its own ability to provoke anti-inflammatory reactions. Kaleido Biosciences is developing KB295 Metabolic Therapy based on the microbiota for mild-to-moderate ulcerative colitis, having successfully completed phase 1 clinical trials [52]. This treatment targets the microbiota directly, focusing on the bacteria’s capacity to use different types of glycans as a substrate for their development [52]. KB295 is a synthetic glycan that selectively supports beneficial bacteria. In the in vitro experiments, this artificial glycan increased short-chain fatty acids selected from Bacteroidetes and Firmicutes, and downregulated Enterobacteriaceae [52]. Overall, KB295 can induce an upregulation of the beneficial bacteria and a downregulation of the harmful bacteria, making it a promising new approach for UC treatment.

**Table 1:** Summary of microbiota associated with health and gut dysbiosis. Note that upward arrows indicate increase and downward arrows indicate a decrease in taxa.

Gut Health and Disease/Dysbiotic Phenotypes	Significant Taxa	Country	References
Normal gut microbiome	↑Firmicutes ↑*Streptococcus* ↑*Veillonella* ↑*Clostridium* ↑*Faecalibactrium prausnitzii* ↑*Blautia faecis* ↑*Roseburia inulinivorans* ↑*Ruminococcus torques* ↑*Clostridium lavalense* ↑Bacteroidetes ↑Proteobacteria	Korea Germany/Lithuania/India	[3,55,56]
Ulcerative colitis	↑Proteobacteria ↑*Escherichia* ↑*Klebsiella* ↑*Bacteroidetes* ↓Firmicutes ↓ *Roseburia* ↓ *Faecalibacterium* ↓ *Eubacterium hallii* ↓ *Gemmiger formicilis* ↓ *Eubacterium rectale* ↓ *Ruminococcus bromii* ↓Tenericutes	Netherlands Italy China Germany/Lithuania/India	[33,35,38,43,56,57,58]
	↓Actinobacteria ↓*Bifidobacterium longum*	Netherlands	[58]
	↓Cyanobacteria ↓Fusobactera ↑Verrucomicrobia	Italy	[59]
Chron’s disease	↑Fusobacteria ↑*Fusobacteriaaceae*	Southern China Israel	[17]
	↓Firmicutes ↓*Eubacterium rectale* ↓*Faecalibacterium prausnitzii* ↓*Roseburia intestinalis* ↓*Roseburia inulinivorans* ↓*Blautia faecis* ↑Bacteroidetes ↑*Bacteroides fragilis*	Netherlands Korea Germany/Lithuania/India	[55,56,58]
*Clostridioides difficile infection*	↑*Clostridioides difficile* ↓*Bacteroidetes* ↓*Bacteroides* ↓Firmicutes ↓*Lactobacilllus* ↓*Enterococcus* ↓*Bacillus* ↓*Faecalibacterium* ↓*Ruminococcus*	France	[60]
Obesity	↓Actinobacteria ↓*Bifidobacterium longum* subsp. *longum* ↓*Bifidobacterium bifidum*	Italy Brazil	[61,62]
	↑Firmicutes ↑*Eubacterium* ↑*Roseburia* ↓*Faecalibaterium* ↓*Clostridiaceae* ↑*Bacteroides*	Japan Korea Mexico	[63,64,65,66]

## 3. *Clostridioides Difficile*—A Special Case of Antibiotic-Driven Dysbiosis

*Clostridioides difficile* is a Gram-positive anaerobic bacterium that forms spores associated with gastrointestinal infections. It is one of the most pressing threats to human antimicrobial resistance and gut inflammation [43]. The gut microbiome is significantly impacted by *C. difficile*, a member of the Firmicutes phylum. *C. difficile* spores are tolerant to extreme environments and utilize this to colonize disrupted or altered microbiomes [67]. This bacterium has two lethal toxins [Enterotoxin (Toxin A, tcdA) and Cytotoxin (Toxin B, tcdB)] that together are the causative agents of *C. difficile* infections in the gut [68,69,70]. *Clostridioides difficile* infections develop due to the inflammation and cellular events these toxins produce [71,72]. Up until 2017, CDI-related hospitalizations were continuously increasing in healthcare- and community-associated infections in the United States [73]. A study of the burden of *C. difficile* infections showed that healthcare-associated infections were higher than community-associated infections and antibiotic exposure, the most common risk factor for acquiring *C. difficile* infections [74]. CDI can cause a variety of symptoms, from intense diarrhea to chronic infections known as inflammatory bowel diseases (IBDs), underscoring the gravity of this condition [75]. Colonization resistance is crucial for the gut microbiota in the fight against pathogens and opportunistic bacteria like *C. difficile* [76]. A balanced gut with protective bacteria consists of taxa such as *Bacteroides*, *Lactobacillus*, *Enterococcus*, *Bacillus*, *Faecalibacterium*, and *Ruminococcus* [60]. Dysbiosis has several community profiles but can be mostly identified as a decrease in the probiotic bacteria. The reduction in alpha diversity and an increase in *C. difficile* infections have been related to the development of IBD illnesses such as ulcerative colitis (UC) and Crohn’s disease. Lifestyle changes, mostly diet, can alter the gut microbiota as previously mentioned [71,76]. Dysbiosis in the gut through the colonization of *C. difficile* is facilitated by toxins A and B by proliferation and disruption, causing diarrhea and diarrhea-like symptoms [73]. A recent study in 2024 confirmed that lower alpha diversity is a risk factor for CDI. This study found a significant association between individuals with a history of CDI and their stature (CDI patients were shorter) compared to those without CDI history, and this association was mediated by alpha diversity [77].

## 4. Inflammation Is a Matter of Microbial Dysbiosis Resulting in Obesity

**Figure 2:**
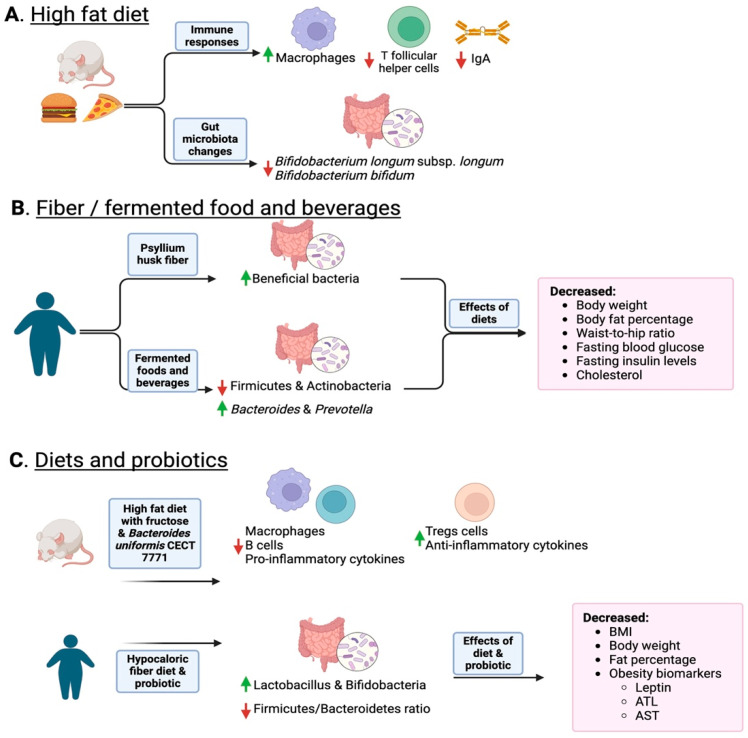
Overview of how diet and the consumption of fiber and fermented foods change the gut microbiota. Panel (**A**) shows how a high-fat diet induces a reduction in IgA and permeability as well as a reduction in probiotic Bifidobacteria. Panel (**B**) shows how fiber and fermented foods such as kimchi increase protective bacteria and lean phenotypes. Panel (**C**) shows how combining diet and probiotics helps decrease inflammation and obese phenotype markers. In the figure, upward green arrows indicate an increase in specific taxa, while red downward arrows indicate a decrease. Created with Biorender.

Obesity is a matter of microbes and their associated inflammatory pathways. Research conducted at Washington University in St. Louis demonstrated that obesity can be transferred with fecal matter [78]. This incredible and simple experiment showed how stool samples from an obese twin, placed into the gut of a gnotobiotic mouse, transferred the obese phenotype to the animal. The same occurred with fecal matter from the lean twin [78]. Obesity might even be considered to be an infectious disease. It was originally expected by 2022 for obesity to affect nearly 890 million adults and 160 million children [78], and a recent update declared that between 2020 and 2035, the prevalence of severe obesity in richer communities is predicted to double from 10 to 20%, posing a serious danger to healthcare systems [79]. Obesity may differ with ethnicity and correlate with socioeconomic status as well as changes in diet, such as high-fat diets [80]. Obesity is thus associated with an inflamed gut, fluctuation in the gut microbiota, and developing diseases such as diabetes, cardiovascular diseases, cancer, and digestive disorders [81,82]. Obese patients have been found to have a reduced abundance of *Bifidobacterium longum* subsp. *longum*, which contributes to glucose and lipid metabolism, and *Bifidobacterium bifidum*, which contributes to decreasing weight and insulin resistance [61]. For type 1 diabetic patients, an increase in *Bifidobacterium adolescentis* has been found [61] and has been suggested to be implemented in diets for nutritional modifications for these diseases. Pro-inflammatory responses in obese individuals may affect gut permeability, and the effect of high-fat diets associated with the intake of processed foods leads to increased levels of pro-inflammatory macrophages [81]. Adding *Bacteroides uniformis* CECT 7771 has been shown to help regulate mice’s responses. Mice given a high-fat diet supplemented with fructose and administered *B. uniformis* were found to reduce body weight gain and restore immune cells by reducing B cells and macrophages and increasing Tregs; the pro-inflammatory cytokines decreased to increase anti-inflammatory cytokines [83] (Figure 2A,C). Lastly, the expression of Toll-like receptor 5 (TLR5) was restored to regular expression levels. TLR5 is important for metabolism since its deficiency can lead to hyperlipidemia, insulin resistance, weight gain, and changes in gut microbiota [83,84]. An essential component of inflammation in obesity is a T cell-mediated immune response (Figure 2). Previous research in pre-clinical models showed how a deficiency in the innate adaptor molecule Myd88 leads to impaired development of T follicular helper cells and reduced IgA production in the gut, subsequently impacting the microbiota and leading to a metabolic syndrome [63]. A study observed that T-Myd88^-/-^ mice would increase weight with aging and were more prone to develop obesity. Compared to a high-fat diet, it would accelerate the development of a metabolic syndrome. To ensure that microbiota is an influential factor in T-Myd88^-/-^ mice in the high-fat diet, they were treated with antibiotics, and it was realized that the mice did not increase in weight [85]. Another observed factor was that T-Myd88^-/-^ mice in the high-fat diet had lower microbial diversity in the ileum and fecal contents, specifically *Clostridiaceae*. Later, in mice enriched in *Clostridiaceae* and *Lachnospiracea*, it was found that when *Desulfrovibrio* colonization occurs, there was a reduction in *Clostridium* that led to weight gain. Lastly, it was demonstrated that mice Tcrb^-/-^ (essential in T cell function) given T-Myd88^-/-^ CD4+ T cells, gained a lot of weight, and a low percent of bacteria were coated by IgA compared with the Tcrb^-/-^ mice given WT CD4+ T cells [63,64]. This emphasizes that the development of T follicular helper cells is essential for IgA production in the gut. IgA production is another essential component for the gut microbiota, which decreases in a high-fat diet. It has been demonstrated with a mice model that plasma cells producing IgA and B cell IgA were reduced during a high-fat diet, thus decreasing the amount of immunoglobulin. IgA deficiency in mice leads to increased T cells, which means inflammatory responses. Lastly, the IgA-deficient mice were observed to have major intestinal permeability and microbes closer to the epithelial cells with high endotoxin levels in the serum, establishing the importance of IgA for the mucous layer [86]. IgA’s significance in the gut is in its prevention of the flagellated bacteria from getting to the epithelial cells. A study demonstrated that mice administered a high-fat diet and flagellin immunized showed decreased intestinal inflammation [87], indicating flagellin—a potent immunomodulatory agent—was as a possible way to reduce inflammation (Figure 2). Table 2 compares the dysbiosis in different phenotypes in both mice and humans.

## 5. Restoration Procedures for Gut Dysbiosis

The emergence of dysbiosis and gastrointestinal diseases, including the inflammatory patterns discussed above or the antibiotic-driven *Clostridioides difficile* infections, has spurred the development of therapies and strategies to maintain healthy gut flora and combat these infections. Treatment with antibiotics like Vancomycin and Fidaxomicin [105] has been the most successful at fighting inflammatory bowel diseases and *C. difficile* infections, but these therapies have not been entirely successful at treating *C. difficile* spores [105]. It is important to note that antibiotic therapies for CDI are strongly associated with increased antibiotic resistance and a less diverse bacterial community in the gut [106], highlighting the potential risks and the need to consider their use with utmost care and caution.

### 5.1. Fecal Microbiota Transplantation (FMT)

Fecal microbiota transplantation (FMT) is a promising approach to restore the gut microbiome and reduce *C. difficile* colonization [75]. The first study back in 2010 reported treating a patient with chronic diarrhea with the fecal matter from a healthy donor (husband) for a successful restoration [107]. This first report shows how natural fecal microbial communities positively impact the balance of the gut microbiome [107]. This method involves the transfer of stool from a person with a healthy diverse microbiota to a patient who presents intestinal dysbiosis or IBD symptoms. It has been found in some cases to treat and reverse *Clostridioides difficile* infections that are resistant to antibiotics [108,109]. The donor’s beneficial microbes potentially colonize the damaged mucosa of the patient to regulate intestinal flora and start to reverse dysbiosis (Figure 1B). A study conducted at Tongji Pediatric Hospital in child Crohn’s disease patients demonstrated that the combination of FMT with partial enteral nutrition (PEN) resulted in higher clinical remission rates, lower CRP inflammation markers, mucosal healing, and disappearance of ulcers in comparison to patients who only underwent PEN therapy [109].

Other studies have shown that FMT can lower antimicrobial resistance and inhibit strains of *C. difficile*. With the continuous improvement of FMT as a treatment for *C. difficile* infections, donor selection has also been an emerging topic. The concept of the “super-donor” is being used to describe those whose donations have been highly successful for patients [110]. Even though FMT for microbiota restoration is simpler for CDI patients, there is still ongoing research on the factors that influence the super-donor stool samples, such as microbial and genetic interactions [110]. This innovative treatment, approved by the Food and Drug Administration (FDA) in 2022, can significantly improve the management of CDI, offering a ray of hope in the fight against this infection. The first biotherapeutic drug, REBYOTA (RBL), consists of live microorganisms from the Bacteroidetes and Firmicutes phyla collected from healthy human stool samples [111,112]. In a randomized, placebo-controlled, pivotal phase 3 trial, the drug’s efficacy and safety analysis demonstrated that RBL improves microbiome diversity and prevents *C. difficile* infections at increasing rates [80]. Another recent and approved therapy is the spore-delivering SER-109 drug, which consists of bacterial spores of healthy individuals and is used to combat recurrent *C. difficile* infection (rCDI) [113]. It was demonstrated that SER-109 administration in high-risk patients of rCDI increased Firmicutes bacteria and decreased *C. difficile* spores [113]. Another study that supports the efficacy of live fecal microbiota oral therapy determined that administration of this drug significantly reduces rCDI when compared with control groups with recurrent infections [114].

### 5.2. FMT Safety Concerns and Alternative Therapies

FMT is typically regarded as safe, particularly when thorough donor screening and testing are performed. Most immediate risks are related to the colonoscopy procedure if involved [115]. The long-term risks are not as well understood, but ongoing follow-up of treated patients is being conducted to monitor this, with few complications reported [115]. Among the most common risks are the following: (1) transmission of pathogens, including resistant bacteria, infectious agents, viruses, and parasites, previously not identified from the donor to the recipient; (2) abdominal discomfort irritation and adverse cramping, which range from mild gastrointestinal symptoms to more severe symptoms; and (3) severe adverse effects leading to inflammatory diseases and even death (less than 1% of patients) [116]. The long-term effects of FMT are not yet fully understood. There is concern about the possibility of transferring not just beneficial but also harmful microorganisms, as well as resistant bacteria that could have unknown long-term consequences, including alterations in the recipient’s microbiome that might increase the risk of chronic conditions such as autoimmune diseases or obesity [115,117].

Regulatory processes can still be considered a little blurry. FMTs in the UK are medicinal products; in the rest of Europe, they are human tissue products, while in the US, they are considered biological products [117]. The lack of standardized methods and regulations creates restrictions and confusion when implementing stool banks, and discourages medical doctors from seeking these innovative therapies [117].

There are also challenges for FMT development, especially the burden of donor screening, the costs associated with pathogen identification in patients, and the difficulty in finding suitable donors. Race and ethnicity account for a statistically significant proportion of the variation in human gut microbiome composition. Even after accounting for all demographic, dietary, and other relevant factors significantly linked to the gut microbiome, the impact of ethnicity on the gut microbiota remained evident [118]. This important variation advocates for and justifies local collections as essential health keepers to deliver the most appropriate stool/FMT from the standpoint of genetics and physiology. Alternatives to FMT that are becoming popular include (1) creating bacterial community consortia designed to mimic healthy communities to overcome transmission of pathogens [119]; (2) delivery of small drugs such as reactive sulfur species (R-SSH) that can selectively modulate the gut microbiome [120]; and (3) phage therapy aimed at targeting bacteria to be eliminated or at neutralizing the expression of a virulence factor [121], as well as probiotics and prebiotic fibers, which we discuss below.

The term probiotic, introduced by Elie Metchnikoff, came about from fermented milk that presented a high abundance of Lactobacilli and was proposed to be a good source of the firmicutes that are beneficial to the GI tract [122]. Although a recent study also showed that probiotic supplementation may delay microbiota restoration compared to fecal matter transplants [123], many studies still show the improvement of gut microbiomes through probiotic consumption. Probiotics have been demonstrated as a restoring therapy against recurrent *Clostridioides difficile* infections and antibiotic resistance [76], and have also been useful for inflammatory diseases, as discussed above, as well as in obesity. Living microorganisms of the gut flora, such as *Lactobacillus acidophilus*, *Lactobacillus casei*, *Saccharomyces boulardii*, and *Bifidobacterium* species [124], are suggested for the administration of probiotic therapy against *C. difficile* [125]. In a study of the suppression of *C. difficile* with the use of *Bifidobacteria*, it was shown that the synthesis of oligosaccharides to grow beneficial bacteria such as *B. infantis* inhibited *C. difficile* in mice. Probiotic strains, such as *Lactocaseibacillus rhamnosus* ATCC 53103 and *Limosilactobacillus reuteri* have been well studied, providing good evidence against abdominal pain and IBS [126], as recently mentioned in the World Gastroenterology Organization’s Probiotics and Prebiotics Guidelines [127].

Prebiotics, which are often cheaper alternatives to probiotics, are being suggested as great fiber sources, or sources of good natural microbial communities that can select for fermentative microbes. Gut microbiota composition is altered due to nutrition changes, and it has been proven that healthy diets that select beneficial bacteria like *Bifidobacteria* and *Lactobacillus* promote a healthier gut environment [128] (Table 3).

Prebiotics are mostly non-digestible foods that promote the growth and activity of beneficial microorganisms, as well as healthy dietary fibers that select for fermentative bacteria. Prebiotics have the capacity to suppress the growth of harmful bacteria and promote the growth of beneficial bacteria, thereby helping to restore the balance in the gut microbiome [129] (Figure 1B). One such prebiotic is stachyose, which is extracted from plants. Studies have shown that this prebiotic can decrease the presence of inflammatory cytokines and reduce the shortening of the colon in mice [130]. Other prebiotics, such as fructooligosaccharide (FOS) and galactooligosaccharide (GOS), have the capacity to induce a positive selection in genera like *Faecalibacterium*, *Lactobacillus*, and *Bifidobacterium* [131]. These genera have been previously described as beneficial for the host and downregulated in UC. Moreover, FOS prebiotics can reduce oxidative stress, possess antioxidant activity, and contribute to overall gut health. On the other hand, GOS can modulate immune function, increasing anti-inflammatory cytokines [131]. Overall, these prebiotics induce a positive regulation of the beneficial bacteria and possess other functions that maintain intestinal integrity and homeostasis. Treatment strategies based on probiotics and prebiotics previously described as beneficial or inhibiting metabolic pathways for the over-expressed metabolites could offer promising options for this illness [131,132].

A fiber-rich diet may include the consumption of psyllium, a water-soluble fiber that improves intestinal flora. Abdominal discomfort with intense epigastric pain can be improved with psyllium husk fiber and lifestyle modifications [132]. The implemented lifestyle modifications include diet (consuming more fruits and vegetables and reducing sugar intake) and physical activity (30 min walks). The study compared gender-wise groups, showing that females improved their symptoms [132] (Figure 2B). Another study supported the use of psyllium husk fiber to improve the microbial composition of constipated patients. It was observed that the use of fiber increased beneficial microbiota, including three butyrate-producers: *Lachnospira*, *Roseburia*, and *Faecalibacterium* [133].

Fermented foods are special cases of prebiotic supplementation. A study in Korea demonstrated the effects of kimchi on obese women. Women moved from having fewer Firmicutes and *Actinobacteria* and higher levels of *Bacteroides*, to higher levels of *Actinobacteria* after kimchi consumption [65] (Table 3). Indeed, after eight weeks of administering kimchi, clinical parameters, such as body weight, body fat percentage, and cholesterol decreased, with the exceptions of triglyceride and pulse rate [65]. In another study, the group was gender-matched and results confirmed that the intervention of fresh kimchi or fermented kimchi for 4 weeks had the ability to decrease weight, BMI, and body fat [134]. The administration of fermented kimchi reduced waist-to-hip ratio, blood glucose, and insulin levels [134]. A hypocaloric fiber regimen combined with physical exercise and probiotic administration was used in a group of obese women [135]. Fiber diet and probiotic administration increased the abundance of protective *Lactobacillus* and *Bifidobacteria* and reduced the ratio of Firmicutes/Bacteroidetes (Figure 2C). Similar research on obese people with type 2 diabetes, after supplementation with specific strains of *Lacticaseibacillus paracasei and Bifidobacterium breve* as probiotics, and galactooligosaccharides as prebiotics, showed there was a rise in the abundance of protective Bifidiobacteriaceae [136]. In obese children and adolescents, a synbiotic composed of *Lactobacillus coagulans* SC-208 and *Lactobacillus indicus* HU36 coupled with a prebiotic short-chain fructooligosaccharide resulted in a decrease in the waist–height ratio [137]. The use of probiotics and prebiotics in obese people suggests that this is an important factor in modulating the gut microbiota to restore the beneficial bacteria and enhance the reduction in weight, BMI, or body fat.

Yogurt is another known probiotic implemented in everyday diets that may modulate the gut microbiota [138]. A specific case of a commercial yogurt—known as 166 yogurt by Sanyuan Foods Co.—was designed to contain fermented milk with a culture of *Lactobacillus bulgaricus* and *Streptococcus thermophilus* with an additional supplementation with *L. acidophilus*, *L. plantarum*, *L. paracasei*, and three *Bifidobacterium strains: breve*, *lactis*, and *longum* (Table 3). To this probiotic mix, they also added fructooligosaccharides, inulin polydextrose, galactooligosaccharide, isomaltooligosacharides, and xylooligosaccharides. This yogurt symbiotic was demonstrated to improve constipation in mice and humans [138]. This improvement in alleviating the constipation symptoms may be attributed to the metabolites that are produced by the bacteria, through stimulation of the prebiotic fibers [138]. In another study, the effect of fresh yogurt and heat-treated yogurt was assessed on immune function in healthy individuals. Fresh yogurt was demonstrated to have a better effect on the immune system since it stimulated the synthesis of IFN-gamma, phagocytic cells, proinflammatory cytokines, and lymphocytes such as Th1 that contribute to IFN-gamma synthesis. The heat-treated yogurt demonstrated an increase in NK cells, Th2 lymphocytes in response to IL-5, and B cells [139].

Fermented beverages are also known to be important items for gut-diversity-focused dietary plans. Kefir is a milk beverage fermented by yeasts and bacteria. This beverage supplies an ample community of more than 100 lactobacilli species, allowing a resistance response to gut colonization by pathogenic bacteria [140]. Among the microbes present, species of *Lactobacillus*, *Bifidobacterium*, *Acetobacter*, *Streptococcus*, *Candida*, and *Saccharomyces* are mainly abundant [140,141] (Table 3). Most notably *Lactobacillus kefiranofaciens* and *Lactobacillus* kefiri, which are only found in this fermented milk, demonstrate a strong capability to inhabit the gut and a capacity to modulate the intestinal microbiome [141]. Even if the quality and microbial composition of Kefir differs by production and respective brands, research suggests it still possesses the capabilities to alleviate weight gain and support metabolic processes while under Western high-fat diets [142]. A recent clinical carried out at Mayo Clinic Rochester with 53 critically ill patients in an ICU provided Kefir supplementation to treat gut dysbiosis despite antibiotic treatment [143]. Results demonstrated a decrease in microbial diversity due to antibiotic treatment; however, the Gut Microbiome Wellness Index (GWMI), indicating the overall health of the gut microbiota, was shown to increase exponentially after 72 h of Kefir implementation, reducing pathogenic bacteria and easing dysbiosis in a short period [143]. The findings also suggest Kefir’s anti-cancerous, anti-oxidant, and anti-bacterial functions, which are further enhanced by fermentation modulation [144]. As such, studies demonstrate a significant role of the benefits of Kefir products for a variety of health profiles in restoring gut microbial diversity and granting whole body benefits.

Another beverage that is currently being added to diets is Kombucha. This fermented tea consists of sugar, tea leaves, and previous fermentation [145,146,147], which leads to a symbiotic culture of yeast and bacteria that act as prebiotics and probiotics [145,146,147,148]. A study conducted in 2020 demonstrated that Kombucha, as a beneficial and antioxidant drink, needed to be further analyzed due to the findings of the benefits depending on the fermentation type and tea being used [147].

Kombucha can be prepared with any type of tea. Chemical profiles of these teas show that green tea has the highest antioxidant activity compared to white, red, and black teas. In its fermentation period, the tea also grows healthy bacteria and yeast, such as *Acetobacter* spp. and *Gluconobacter* spp. [145,147,149], *Zygosaccharomyces* spp. [145,149], and *Schizosaccharomyces pombe* [147,149] (Table 3). To demonstrate Kombucha’s benefits for healthy diets, a pre–post intervention study revealed that obese individuals who consumed fermented tea showed a decrease in insulin in their bodies [145]. Dietary habits, such as increasing vegetable consumption, complemented with Kombucha, have also been studied in studies aimed at reducing levels of cholesterol. In New Zealand, a study divided rabbits into groups with high-cholesterol diets and others with high-cholesterol diets with Kombucha treatment, and intestinal bacteria were analyzed through stool collection [150]. This study concluded that the Kombucha treatment reduced the high cholesterol levels [150]. Kombucha treatment has also been used for non-alcoholic fatty liver disease in mice. In 2018, Jung et al. demonstrated that mice fed with Kombucha tea had decreased levels of *Clostridium* and reduced fat accumulation compared to groups that did not receive the treatment [151]. Kombucha tea-fed groups also showed increased beneficial bacteria like *E. coli*, *Bacteroidetes*, and *Lactobacillus* [151]. Due to its antioxidant and symbiotic benefits, Kombucha has been a popular drink implemented into multiple diets for its anti-inflammatory effects and reduction in obesity risks and secondary diseases such as diabetes [145]. Spent coffee grounds are also an emerging prebiotic [152]. In vitro studies have shown that mannanoligosaccharides from coffee grounds stimulate the growth in beneficial bacteria such as *Bifidobacterium*, *Ruminococcus*, and *Blautia*, and short-chain fatty acids [153], thus showing the prebiotic activity in coffee grounds and the potential effects on glucose metabolism [153]. Lastly, two far less popular fermented beverages are mauby (made of woody plants) and Tepache (fermented from pineapple rinds), which are traditional drinks in the Caribbean. A pilot study revealed that these drinks are rich in beneficial bacteria, particularly from a diverse array of multi-strain species of *Lactobacillus* and other probiotics. The relative abundance of probiotic changes depending on the source material used for mauby fermentation (different species of wood) [154]. A study of fermented beverages in Mexico demonstrated that Tepache contains lactic bacteria such as *E. faecium* and *L. lactis*. Although more studies are needed, this characterization presents the potential of this drink as an antimicrobial and a prebiotic [155]. Among fruits, kiwi stands out as a major prebiotic fiber source, influencing gut microbiota profiles towards an enrichment of *Lactobacilli* and *Bifidobacteria* (as compared to the same individual before consumption) [156]. Other fruits, such as black raspberries, have also been demonstrated to have prebiotic compounds [157]. In 2019, Gu et al. fed mice black raspberries and analyzed the mice for colon microbiome composition [158]. Mice showed greater Bacteroidetes levels and lower levels of Firmicutes, such as *Clostridium* species, in the gut’s lumen after the raspberry diet. This study also confirmed the improvement in metabolite levels in the gut after the fiber-rich fruit diet [158]. Overall, many fruit and fermented foods are sources of important fermentative taxa that can improve gut biodiversity and anti-inflammatory pathways.

**Table 3:** Summary of probiotics/prebiotics and protective taxa associated with improved gut health outcomes.

Probiotic/Prebiotic	Functions	Outcomes	References
Psyllium	Increased levels of *Lachnospira*, *Roseburia*, and *Faecalibacterium*	Improved abdominal discomfort, epigastric pain, and constipation symptoms	[132,133]
Kimchi	Increased levels of *Actinobacteria*	Improved cholesterol levels, insulin levels, body weight, body fat percentage, and BMI	[65,134]
*Lacticaseibacillus paracasei YIT 9029* and *Bifidobacterium breve YIT 12272*	Increased levels of Bifidobacteriaceae and *Lactobacillus*	Improved glucose metabolism	[136]
Yogurt	Increased levels of *Bacteroides*, *Streptococcus*, *Blautia*, and *Saccharomyces;* NK cells, B cells, IL-5, and Th2	Improved constipation symptoms and immune responses	[138,139]
Kefir	Increased levels of *Lactobacillus*	Improved constipation symptoms, cholesterol levels, and obesity risks	[141,142,143]
Kombucha	Increased levels of beneficial *E. coli*, Bacteroidetes and *Lactobacillus*	Improved glucose levels, obesity risks, and cholesterol levels.	[145,150,151]
Kiwi	Increased levels of *Lactobacilli*, and *Bifidobacteria*	Improved the growth of intestinal lactid acid bacteria and perturbation of *Clostridium*	[156]
Black raspberries	Increased levels of Bacteroidetes	Improved colon microbial α-diversity	[157,158]
Coffee spent grounds	Increased levels of *Bifidobacterium*, *Jamie Quinton*, and *Ruminococcus*	Improved levels of microbial α-diversity in fecal samples	[152,153]
*Bifidobacterium animalis* and *Lactobacillus paracasei*	Increased levels of Bacteroidetes	Improved gut microbiota and risk for metabolic disorder	[159,160]

## 6. Conclusions/Summary

The development of sequencing technologies, improvement in bioinformatic pipelines, and the spread of knowledge on computational methodologies has allowed microbial ecologists and other health experts to survey the global human microbiome. Factors such as poor diet, antibiotic use, infections, and stress can lead to dysbiosis, characterized by a loss of beneficial microbes, an overgrowth of harmful microorganisms, or a loss of microbial diversity. Dysbiosis, an imbalance in the gut microbiota, disrupts essential processes and contributes to diseases such as ulcerative colitis, Crohn’s disease, and *Clostridioides difficile* infections. Challenges posed by antibiotic-driven dysbiosis, particularly concerning *C. difficile* infections, call for novel therapeutic approaches like fecal microbiota transplantation (FMT) and biotherapeutic drugs. Restoration of a healthy gut microbiome through dietary interventions, probiotics, and prebiotics presents itself as a promising strategy to manage gut-related diseases. The connection between gut dysbiosis and obesity suggests that microbial imbalances contribute to inflammatory pathways and associated conditions such as cardiovascular diseases and diabetes.

New integrative approaches are proposed to combine dietary modifications and emerging therapeutic techniques like FMT and synthetic glycans targeting beneficial bacteria. In conclusion, this review underlines the importance of maintaining gut microbiota balance to prevent and manage various inflammatory diseases, proposing integrative approaches that include dietary modifications and emerging therapeutic techniques like FMT and synthetic glycans targeting beneficial bacteria.

## Figures and Tables

**Table 2:** References of animal and human studies supporting the role of the microbiome in gut dysbiosis.

Gut Health and Disease/Dysbiotic Phenotypes	Animal Studies	References	Human Studies	References
Normal gut microbiome	In mammals, opportunistic bacteria that can thrive in dysbiotic habitats displace the microbes that contribute to a healthy ecosystem, influencing the environmental metagenome; as such, a clear difference in microbial populations is seen between captive and wild animals. In the mouse research model, Firmicutes, Bacteroidetes, Proteobacteria, Deferribacteres, Actinobacteria, Tenericutes, and Verrucomicrobia are shown to be commensal inhabitants of the gut microbiota, with the first three being the most abundant taxa.	[88,89,90,91]	An abundance of microbes such as *Bacteroides*, *Lactobacillus*, *Bacillus*, *Enterococcus*, *Faecalibacterium*, *Bifidobacterium* and *Ruminococcus* spp. are characteristic of a healthy and diverse gut microbiota.	[24,25,60]
Ulcerative colitis	In mice with UC, dysbiosis in their microbiota is closely related to disease pathogenesis and symptomatology. Increased populations of Proteobacteria, Actinobacteria, and *Clostridium* spp. were observed in mice with mucosal inflammation correlating to a UC profile. Enterobacteriaceae taxa, *R. gnavus*, and especially *E. coli* are shown to drive colonization of pathobionts and inflammation in colitis mice models.	[92,93,94]	The interactions of the host and their microbiome, a decrease in Firmicutes, an increase in Proteobacteria, reduction in microbial diversity, and alterations in homeostasis are associated with UC pathogenesis. The connection of fungi with certain intestinal bacteria could lead to developing UC. *S. boulardii* and *C. albicans* are seen to confer positive or negative effects, respectively, depending on the bacterial diversity present in the gut microbiota.	[31,33,34,41,95]
Crohn’s disease	The role of the microbiota can be seen in how the transference of microbes from a dysbiotic animal model can influence a healthy system. The transferrence of an altered microbiota from actively diseased mice induced CD symptomatology in healthy mice. Research by Schaubeck et al. points to Bacteroidaceae, Erysipelotrichaceae, Peptostreptococcaceae and Verrucomicrobiaceae taxa in mice that simulate a CD profile. Specifically, *B. acidifaciens* and *B. sartorii* were found in greater abundance. *Bacteroides* spp. are more prevalent when transferring human microbiota into mice.	[94,96,97]	Reduction in bacterial exposure and lower microbial diversity correlates with increased disease incidence. Increase in Fusobacteriaceae-like taxa is linked with the Crohn’s disease profile. CD is also characterized by a decrease in fungal and bacterial interactions. An abundance of fungi, like *C. tropicalis*, can give rise to more opportunistic pathogenic bacteria.	[17,95]
*Clostridioides difficile*	Antibiotics can diminish microbial communities for prolonged periods and eliminate species that could provide colonization resistance against *C. difficile*. A combination of antibiotics and CDI can further alter the intestinal microbiota and increase mucosal inflammation in mice, demonstrating increased abundance in Proteobacteria and Enterococci and a loss of microbial commensal diversity, such as Enterobacteriaceae. Even after the removal of antibiotics, their dysbiotic microbial communities remain susceptible to recurrence of CDI.	[98,99,100]	Antibiotics are known to generate dysbiosis in the human microbiome; in the same manner, they are heavily linked to the pathogenesis of CDI. Beneficial bacteria such as *Bacteroides*, *Lactobacillus*, and *Ruminococcus*, among related taxa, provide resistance for the gut microbiota against developing CDI in a perturbed intestinal system.	[60,76,101]
Obesity	There exist links between the immune system and the gut microbiome. NOD2, as well as TLR5-deficient, mice are prone to increased adiposity. Dysbiotic microbiota transference into germ-free mice resulted in inflammation and gain in adipose tissue.	[78,102,103]	Diet has been proven to influence the microbes in the gut and modulate the levels of inflammation and obesity in a human host, especially in a Westernized high-fat diet selecting for less beneficial bacteria. The unbalanced ratio between Firmicutes and Bacteroidetes in dysbiosis correlates with increased adiposity, weight gain, and BMI.	[10,102,104]
